# Advanced glycation end products accelerate calcification in VSMCs through HIF-1α/PDK4 activation and suppress glucose metabolism

**DOI:** 10.1038/s41598-018-31877-6

**Published:** 2018-09-13

**Authors:** Yi Zhu, Wen-Qi Ma, Xi-Qiong Han, Ying Wang, Xin Wang, Nai-Feng Liu

**Affiliations:** Department of Cardiology, Zhongda Hospital, School of Medicine, Southeast University, Nanjing, 210009 P.R. China

## Abstract

Arterial media calcification is associated with diabetes mellitus. Previous studies have shown that advanced glycation end products (AGEs) are responsible for vascular smooth muscle cell (VSMC) calcification, but the underlying mechanisms remain unclear. Hypoxia-inducible factor-1α (HIF-1α), one of the major factors during hypoxia, and pyruvate dehydrogenase kinase 4 (PDK4), an important mitochondrial matrix enzyme in cellular metabolism shift, have been reported in VSMC calcification. The potential link among HIF-1α, PDK4, and AGEs-induced vascular calcification was investigated in this study. We observed that AGEs elevated HIF-1α and PDK4 expression levels in a dose-dependent manner and that maximal stimulation was attained at 24 h. Two important HIF-1α-regulated genes, vascular endothelial growth factor A (VEGFA) and glucose transporter 1 (GLUT-1), were significantly increased after AGEs exposure. Stabilization or nuclear translocation of HIF-1α increased PDK4 expression. PDK4 inhibition attenuated AGEs-induced VSMC calcification, which was evaluated by measuring the calcium content, alkaline phosphatase (ALP) activity and runt-related transcription factor 2 (RUNX2) expression levels and by Alizarin red S staining. In addition, the glucose consumption, lactate production, key enzymes of glucose metabolism and oxygen consumption rate (OCR) were decreased during AGEs-induced VSMC calcification. In conclusion, this study suggests that AGEs accelerate vascular calcification partly through the HIF-1α/PDK4 pathway and suppress glucose metabolism.

## Introduction

Vascular calcification, an advanced atherosclerotic pathological process similar to osteogenesis that is involved in the intima or media of blood vessels^1^, is associated with elevated cardiovascular morbidity and mortality in patients with diabetes mellitus (DM) or end-stage renal disease^2,3^. Vascular calcification is an active, complex, and chronic process involving inflammation, oxidative stress, and apoptosis^4–6^. Vascular smooth muscle cells (VSMCs), the main cell type of vascular media, undergo an osteoblastic phenotype transition, leading to arterial media calcification^7^.

Advanced glycation end products (AGEs) are derived from non-enzymatic reactions between sugars and the amino groups of protein and are responsible for serious diabetic complications^8^. Previous studies have demonstrated that AGEs promote the osteoblastic phenotype transition among VSMCs and vascular calcification through several signaling pathways^9^. AGEs interact with the receptor for advanced glycation end products (RAGE) to activate oxidative stress, and reactive oxygen species (ROS) further facilitates AGEs formation^10^. Our laboratory has previously reported that AGEs increase oxidative stress in VSMC calcification^11^ and that Nε-carboxymethyl-lysine (CML), a major ingredient of AGEs, could enhance vascular calcification through CML/ROS/pyruvate dehydrogenase kinase 4 (PDK4) activation^12^. PDK4 is a regulator of cellular energy metabolism and is closely related with vascular calcification^13^. Lee S.J *et al*. reported that PDK4 accelerates vascular calcification through SMAD1/5/8 phosphorylation^14^. However, whether PDK4 participates in AGEs-induced VSMC calcification and the exact molecular mechanisms by which AGEs regulate PDK4 have not been investigated in depth.

It is well known that hypoxia influences the osteogenic trans-differentiation of bone cells^15,16^. Hypoxia-inducible factor-1α (HIF-1α) is a major factor during hypoxia and promotes adaptation during oxygen deprivation^17^. A study by Mokas S *et al*. showed that HIF-1α is a potential target against vascular calcification in high-phosphate environments^18^. HIF-1α has also been shown to be involved in chronic kidney disease^19,20^ and runt-related transcription factor 2 (RUNX2)-related pulmonary arterial hypertension^21^, which was mostly accompanied by the presence of vascular calcification. In addition, several reports have demonstrated that HIF-1α is associated with PDK4. Succinate increases PDK4 expression in a HIF-1α-dependent manner, and HIF-1α could bind to the PDK4 promoter evaluated by luciferase^22^. HIF-1α provides neuroprotection and neurorepair partly through increased PDK4 expression^23^; hypoxia could also induce PDK4 gene expression via estrogen related receptor γ (ERRγ)^24^. Mitochondrial reactive oxygen species (mtROS) may be signal molecules that stabilize HIF-1α, thereby reducing its degradation^25^. Thus, we postulated that HIF-1α may be stimulated by AGEs-induced ROS formation and correlated with PDK4 during vascular calcification.

HIF-1α activation contributes to increased glycolysis in VSMC^26^. Pyruvate dehydrogenase kinases (PDKs) are mitochondrial regulators of glucose metabolism that inhibit pyruvate dehydrogenase (PDH) activity, thereby inhibiting the transition of pyruvate to acetyl-CoA and eventually reducing Kreb’s cycle flux^27^. At present, the roles of HIF-1α and PDK4 in AGEs-mediated VSMC calcification require further exploration, and AGEs-induced glucose metabolism changes remain to be investigated. Under the premise that AGEs up-regulate HIF-1α and PDK4 expression, whether AGEs could enhance glycolysis in VSMC was investigated in this study.

In the present study, we investigated the role and potential mechanisms of the HIF-1α/PDK4 signaling pathway in AGEs-induced vascular calcification through *in vitro* experiments. Our results revealed that AGEs accelerate calcification in VSMCs through HIF-1α/PDK4 activation. Interestingly, AGEs suppress glucose metabolism during the calcification process.

## Results

### Effect of AGEs on VSMC viability during VSMC calcification

**Figure 1:**
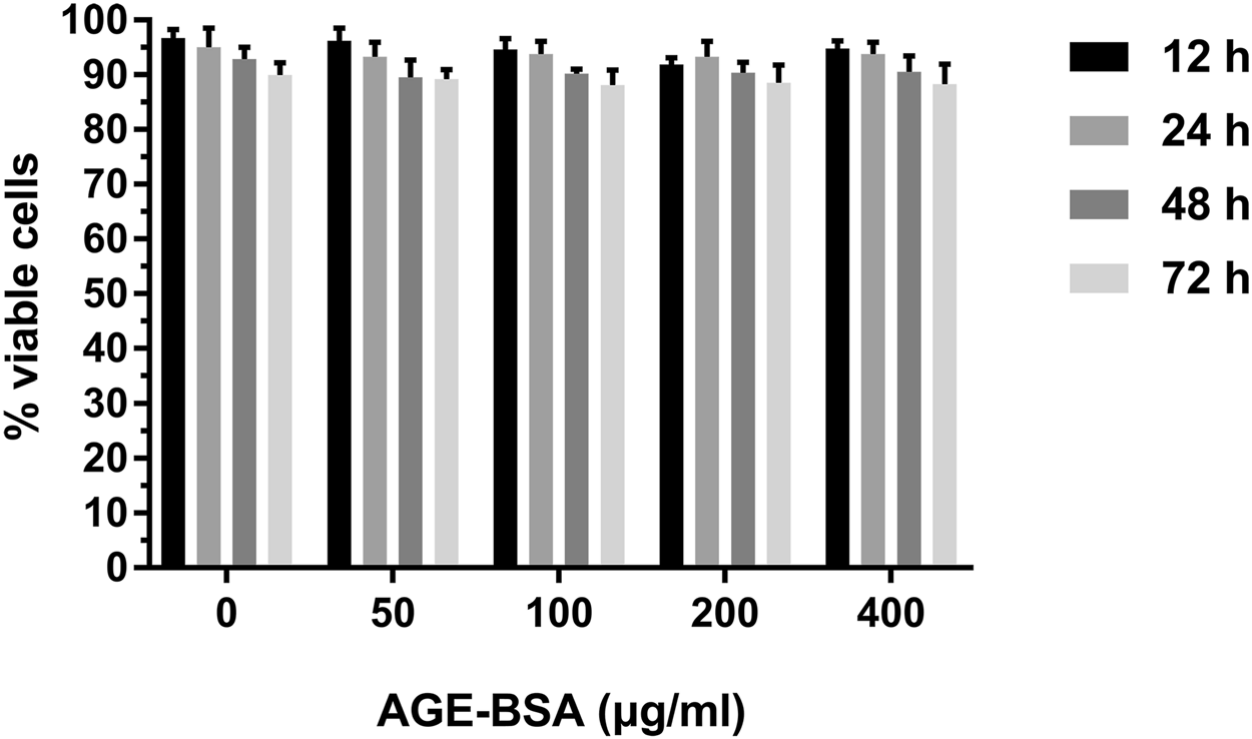
Effects of AGEs on VSMC viability. Calcified VSMCs were cultured with AGE-BSA (0, 50, 100, 200, and 400 μg/ml) for 12, 24, 48, and 72 h. The cell viability was evaluated by CCK-8 assay.

VSMCs were treated with AGE-BSA (0, 50, 100, 200, and 400 μg/ml) in the presence of 10 μM β-GP for 12, 24, 48, and 72 h. Cell viability was evaluated by the CCK-8 assay. Figure 1 shows that 50–400 μg/ml AGE-BSA treatment at different time points had no significant effects on cell viability. For this reason, AGE-BSA ranging from 50–400 μg/ml was applied at the abovementioned durations for subsequent experiments.

### AGEs enhanced HIF-1α and PDK4 expression during VSMC calcification

**Figure 2:**
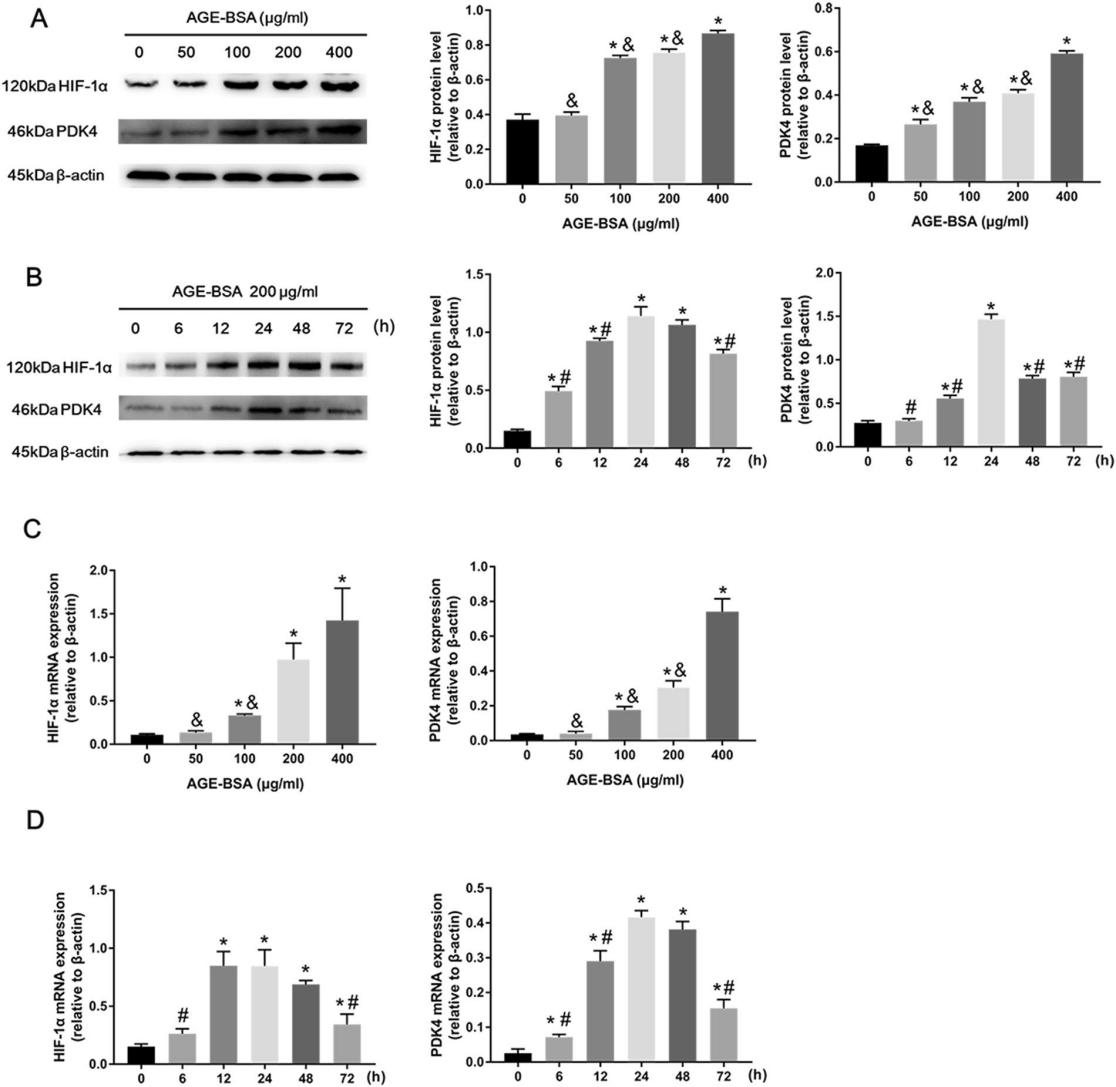
AGEs increased HIF-1α and PDK4 expression. HIF-1α and PDK4 expression in calcified VSMCs treated with AGE-BSA at different concentrations and times were evaluated by western blotting **(A**,**B)** and qRT-PCR **(C**,**D)**. *P < 0.05 compared with the normal control group. ^&^*P* < 0.05 compared with the AGE-BSA (400 μg/ml) group. ^#^*P* < 0.05 compared with the AGE-BSA (24 h) group.

To investigate the effects of AGE-BSA treatment on HIF-1α and PDK4 expression, VSMCs were treated with AGE-BSA (0, 50, 100, 200, and 400 μg/ml) containing 10 mM β-GP for 24 h. HIF-1α and PDK4 protein and mRNA expression levels were determined by western blotting and qRT-PCR. We found that the protein and mRNA expression levels of HIF-1α and PDK4 were significantly increased in a dose-dependent manner (Fig. 2A,C). Then, we incubated VSMCs with AGE-BSA (200 μg/ml) containing 10 mM β-GP for 0, 6, 12, 24, 48, and 72 h. The protein and mRNA expression levels of HIF-1α and PDK4 were analyzed by western blotting and qRT-PCR. We observed that HIF-1α and PDK4 protein and mRNA expression levels were increased in AGE-BSA-treated groups compared with the normal control groups, and this increase was maximal after 24 h of stimulation (Fig. 2B,D). Taken together, these results indicate that HIF-1α and PDK4 transcription and translation are increased during AGEs-induced VSMC calcification.

### AGEs promoted HIF-1α nuclear translocation and HIF-1α-regulated gene expression

**Figure 3:**
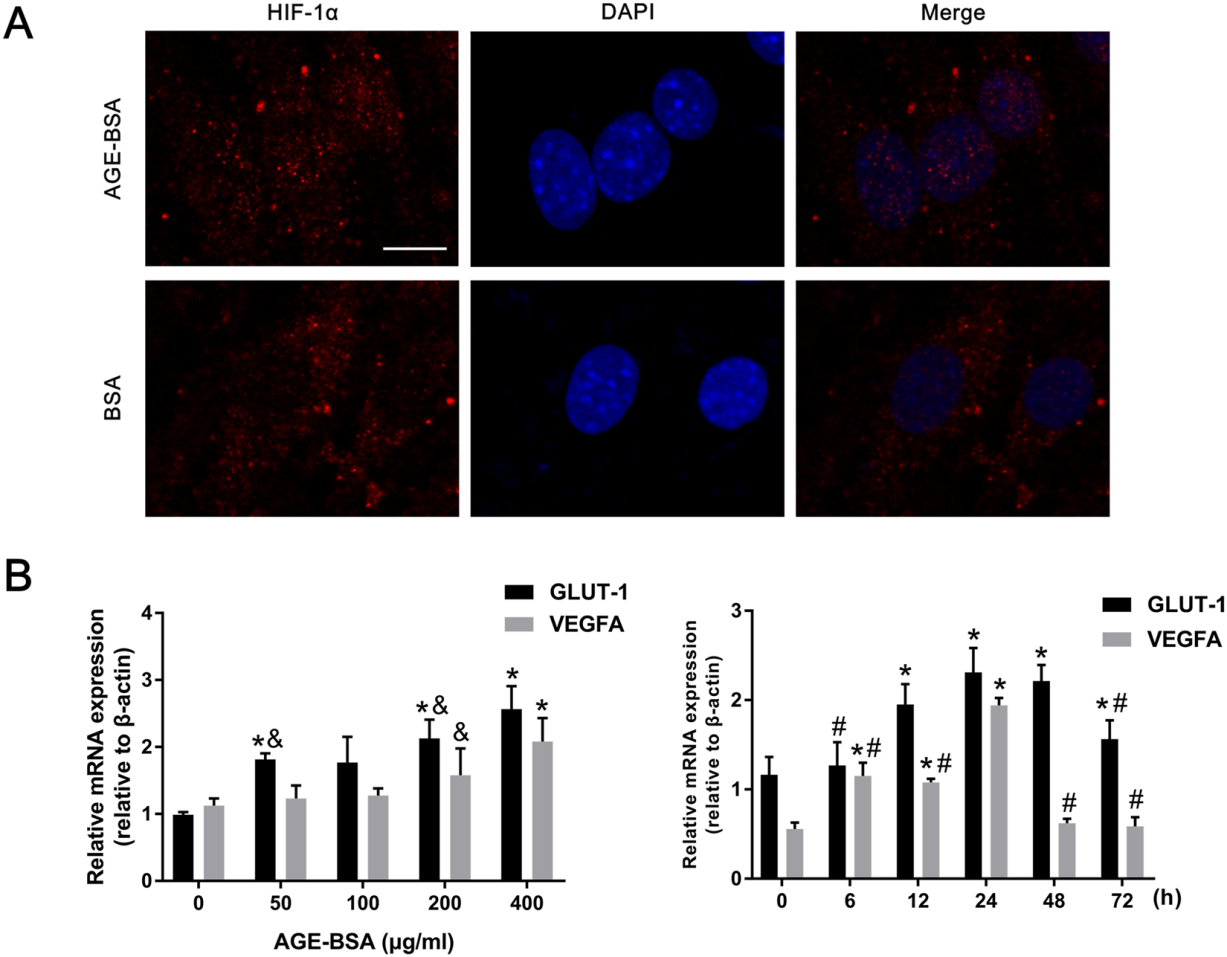
AGEs induced HIF-1α nuclear translocation and HIF-1α target gene activation. (**A**) HIF-1α nuclear translocation in calcified VSMCs after AGE-BSA treatment (200 μg/ml) was visualized by immunofluorescence staining; scale bar: 10 μm **(B)** GLUT-1 and VEGFA mRNA expression in calcified VSMCs treated with AGE-BSA at different concentrations and times were evaluated by qRT-PCR. *P < 0.05 compared with the normal control group. ^&^*P* < 0.05 compared with the AGE-BSA (400 μg/ml) group. ^#^*P* < 0.05 compared with the AGE-BSA (24 h) group.

As mentioned above, HIF-1α expression levels were elevated in VSMCs treated with AGE-BSA. To further demonstrate that increased transcriptional expression of HIF-1α is associated with AGE-BSA treatment, VSMCs were incubated with AGE-BSA or BSA (200 μg/ml) containing 10 mM β-GP for 24 h. HIF-1α nuclear translocation was visualized by immunofluorescence with a confocal microscope. We observed that AGE-BSA significantly promoted HIF-1α translocation into the nucleus compared with the BSA group (Fig. 3A). Western blotting also showed that AGE-BSA significantly elevated nuclear HIF-1α expression (Supplementary Figure 1A), further suggesting that AGE-BSA can promote HIF-1α nuclear translocation.

Since HIF-1α binds to hypoxic response elements (HREs), which are found on target genes such as vascular endothelial growth factor A (VEGFA) and glucose transporter type 1 (GLUT-1)^28^, we next explored the expression levels of two important HIF-1α target genes. We cultured VSMCs with AGE-BSA (0, 50, 100, 200, and 400 μg/ml) containing 10 mM β-GP for 0, 6, 12, 24, 48, and 72 h; interestingly, elevated HIF-1α expression was accompanied by increased HIF-1α target gene expression. Similar to HIF-1α, mRNA expression levels of GLUT-1 and VEGFA were augmented during VSMC calcification (Fig. 3B). Thus, our results suggest that HIF-1α and AGEs are implicated during vascular calcification.

### HIF-1α activated PDK4

**Figure 4:**
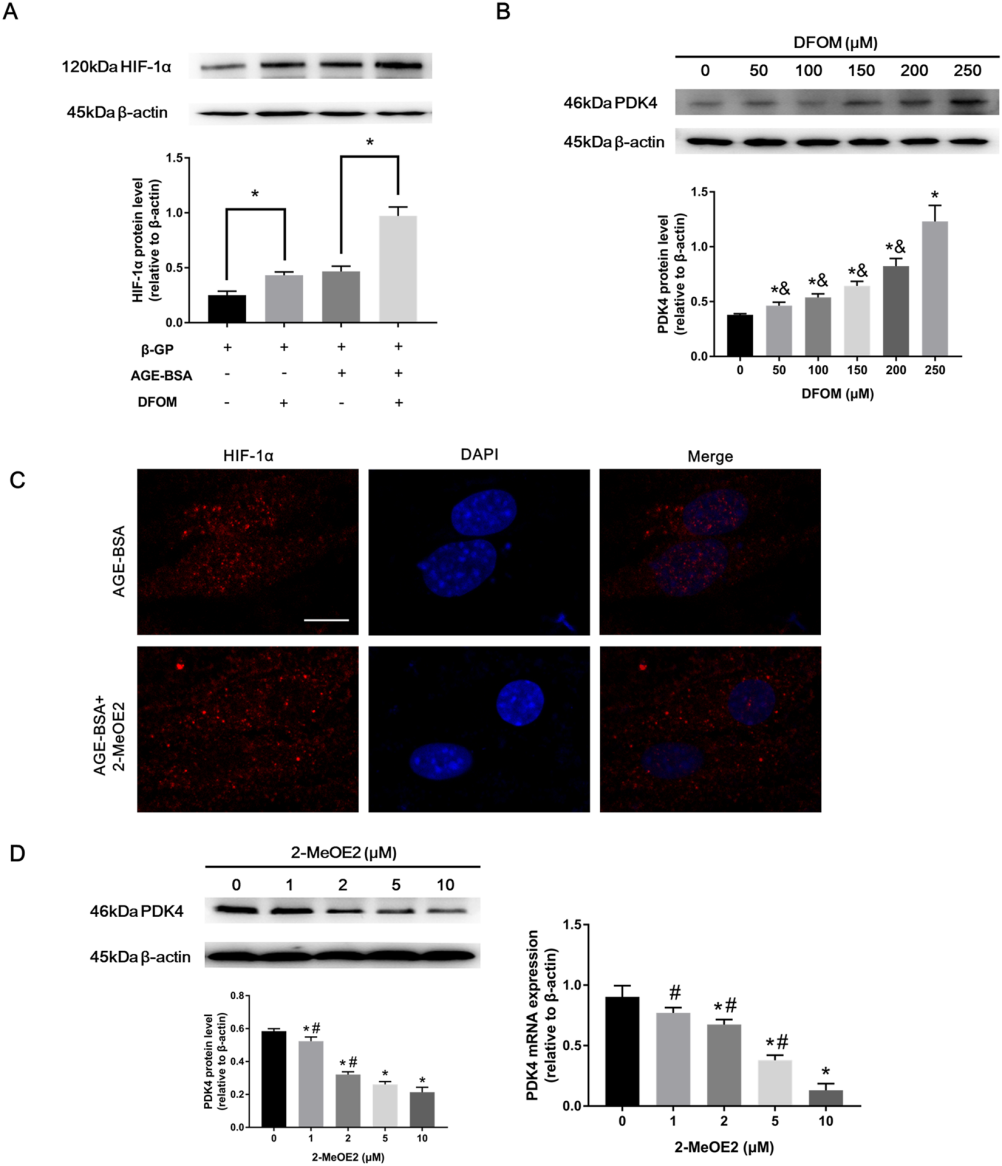
PDK4 is associated with HIF-1α during VSMC calcification. (**A**) Calcified VSMCs were pretreated with DFOM (250 μM) for 6 h and then cultured with or without AGE-BSA (200 μg/ml) for 24 h. HIF-1α protein levels were determined by western blotting. *P < 0.05 vs. the indicated treatment. **(B)** Calcified VSMCs were preincubated with DFOM for 6 h, and the cells were exposed to AGE-BSA (200 μg/ml) for another 24 h. PDK4 expression was detected by western blotting. *P < 0.05 compared with the normal control group. ^&^*P* < 0.05 compared with the DFOM (250 μM) group. **(C)** Calcified VSMCs were pretreated with 2-MeOE2 (10 μM) for 2 h and then incubated with or without AGE-BSA (200 μg/ml) for 24 h. HIF-1α nuclear translocation in VSMCs was visualized by immunofluorescence staining; scale bar: 10 μm **(D)** After 2 h of 2-MeOE2 exposure, calcified VSMCs were incubated as indicated. PDK4 expression was detected by western blotting and qRT-PCR. *P < 0.05 compared with the normal control group. ^#^*P* < 0.05 compared with the 2-MeOE2 (10 μM) group.

Since HIF-1α is involved in vascular calcification and PDK4 has been reported as an important factor in vascular calcification^14^, to investigate whether HIF-1α may participate in PDK4 upstream signaling, we selected DFOM, a hypoxia-mimetic agent to stabilize HIF-1α, and 2-MeOE2, which inhibits HIF-1α translocation to the nucleus. As shown in Fig. 4A, DFOM treatment (250 μM) promoted HIF-1α protein levels compared with the control group. Subsequently, VSMCs were preincubated with DFOM (0, 50, 100, 150, 200, and 250 μM) for 6 h and then exposed to AGE-BSA (200 μg/ml) and 10 mM β-GP for 24 h. As expected, PDK4 protein expression levels were increased in a dose-dependent manner (Fig. 4B), suggesting that HIF-1α stabilization could regulate PDK4 translational expression during VSMC calcification.

We further explored whether HIF-1α transcriptionally regulates PDK4. To confirm that 2-MeOE2 could inhibit HIF-1α translocation, VSMCs were pretreated with 10 μM 2-MeOE2 for 2 h followed by AGE-BSA (200 μg/ml) and 10 mM β-GP exposure for 24 h. HIF-1α nuclear translocation was markedly blocked with 10 μM 2-MeOE2, as detected by immunofluorescence staining (Fig. 4C) and western blotting (Supplementary Figure 1B). To rule out the possibility of 2-MeOE2 causing VSMC calcification, the effects of 2-MeOE2 on VSMC calcification were evaluated, and 2-MeOE2 (10 μM) had no influence on VSMC calcification (Supplementary Figure 2). Then, 2-MeOE2 (0, 1, 2, 5, 10 μM) was applied to inhibit HIF-1α translocation, and the PDK4 expression level was decreased in a dose-dependent manner as evaluated by western blotting and qRT-PCR (Fig. 4D). Thus, these results show that HIF-1α increases the expression level of PDK4 in a transcriptional and translational manner.

### PDK4 knockdown alleviated VSMC calcification

**Figure 5:**
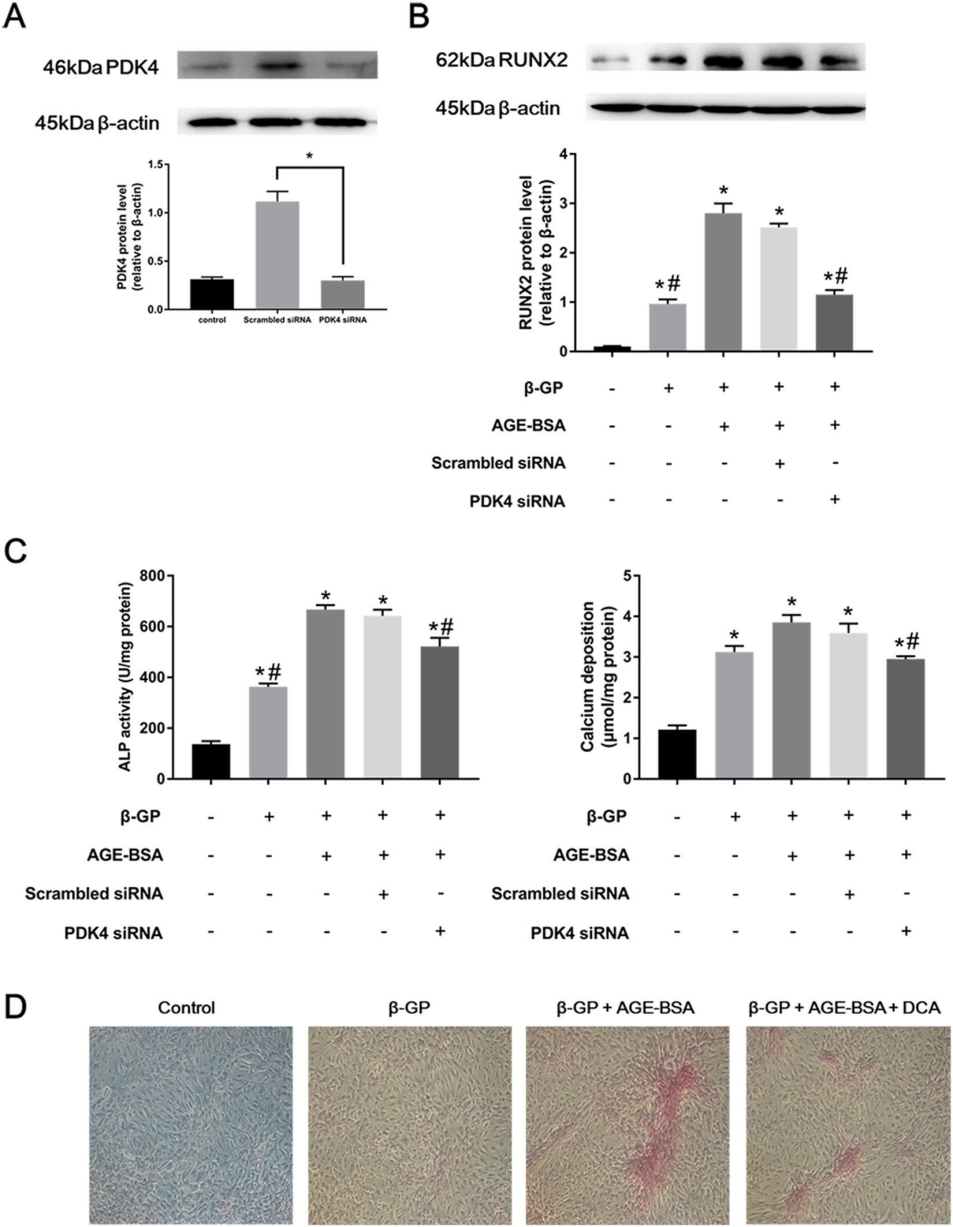
AGEs accelerated VSMC calcification through a PDK4-dependent pathway. (**A**) PDK4 siRNA transfection efficiency was determined by western blotting. *P < 0.05 vs. the indicated treatment. **(B)** Calcified VSMCs were transfected with PDK4 siRNA or scrambled siRNA for 24 h and then cultured with or without AGE-BSA (200 μg/ml) for another 24 h. RUNX2 expression was determined by western blotting. *P < 0.05 compared with the normal control group. ^#^*P* < 0.05 compared with the AGE-BSA +β-GP group. **(C)** After transfection, calcified VSMCs were cultured with or without AGE-BSA (200 μg/ml) for 7 days, and ALP activity and calcium deposition were detected. *P < 0.05 compared with the normal control group. ^#^*P* < 0.05 compared with the AGE-BSA +β-GP group. **(D)** Calcified VSMCs were incubated with or without AGE-BSA (200 μg/ml) and DCA for 21 days. Calcium nodule formation was visualized by Alizarin red S staining.

To explore whether AGEs accelerate VSMC calcification through a PDK4-dependent pathway, small interfering RNA (siRNA) was used for PDK4 knockdown. The knockdown efficiency was nearly 75% after VSMCs transfected with siRNA against PDK4 for 24 h compared with that in the scrambled siRNA group (Fig. 5A). PDK4 siRNA also presented effective transfection efficiency at day 7, although the efficiency was weaker than that observed on the previous 3 days (Supplementary Figure 3). AGE-BSA treatment alone without calcium medium did not obviously cause VSMC calcification and played only an accelerating role (Supplementary Figure 4), and thus, AGE-BSA was used in the presence of β-GP. VSMCs transfected with PDK4 siRNA or scrambled siRNA were cultured in calcium medium with or without AGE-BSA (200 μg/ml). Since RUNX2 is an important factor during VSMC osteoblastic differentiation, we postulated that PDK4 is an upstream molecule of RUNX2. As shown in Fig. 5B, AGE-BSA treatment significantly increased the RUNX2 expression level, as previously reported^12^, and PDK4 knockdown via siRNA down-regulated RUNX2 protein levels, suggesting that the AGEs/PDK4/RUNX2 signaling pathway is present in VSMC calcification. In addition, PDK4 inhibition decreased AGEs-induced ALP activity and calcium deposition content (Fig. 5C). Furthermore, PDK4 inhibition via DCA markedly decreased AGEs-induced calcified nodule formation as shown by Alizarin red S staining (Fig. 5D). All these results reveal that PDK4 plays an important role in AGEs-induced VSMC calcification.

### Effect of AGEs on glucose metabolism changes during VSMC calcification

**Figure 6:**
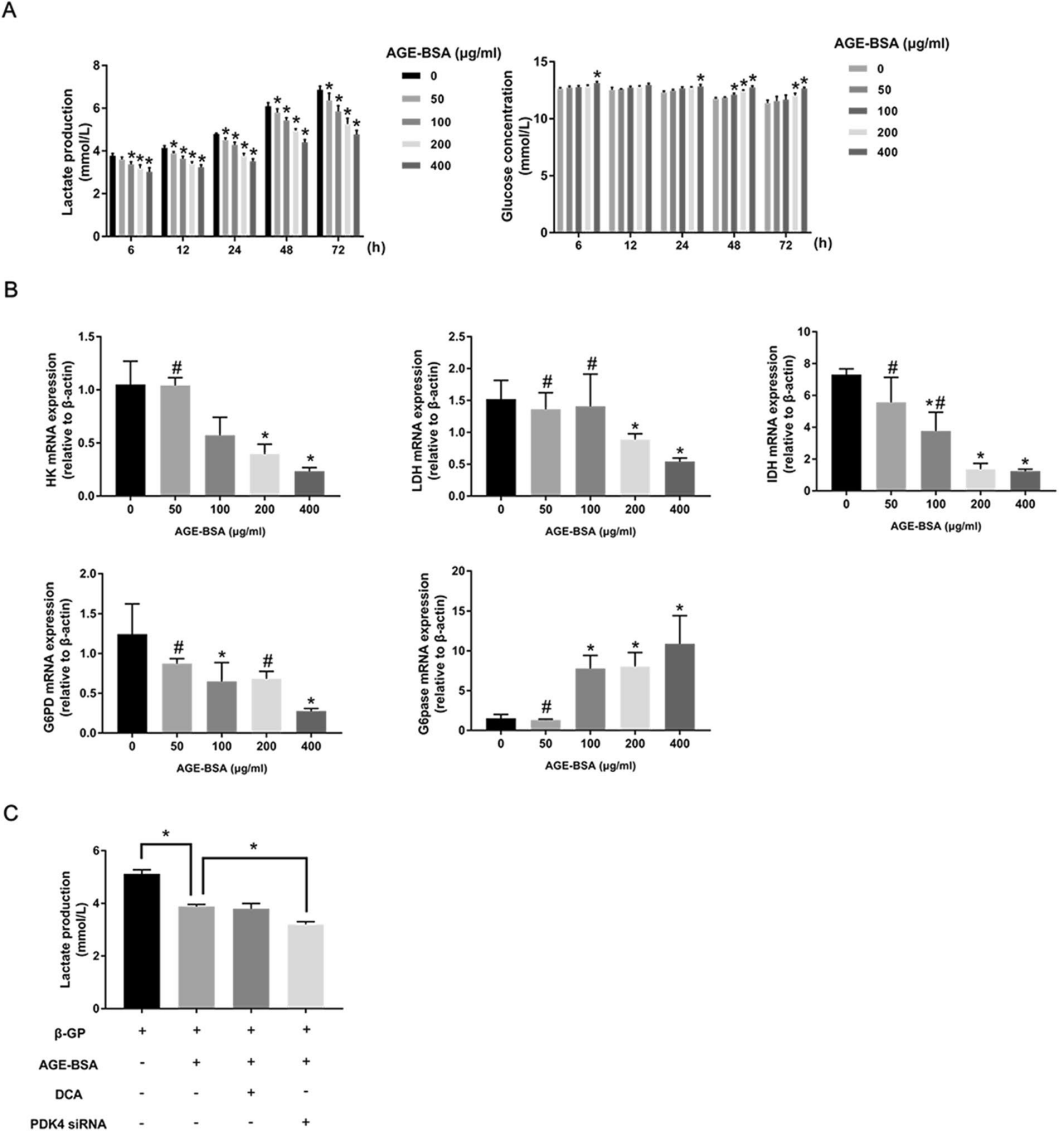
AGEs suppressed glycolysis during VSMC calcification. (**A**) Lactate production and glucose concentrations were analyzed at different time points after AGE-BSA treatment. *P < 0.05 compared with the control group. **(B)** HK, LDH, IDH, G6PD, and G6pase expression levels in calcified VSMCs after AGE-BSA exposure were determined by qRT-PCR. *P < 0.05 compared with the control group. ^#^*P* < 0.05 compared with the AGE-BSA (400 μg/ml) group. **(C)** Calcified VSMCs were pretreated with DCA or PDK4 siRNA and then treated with AGE-BSA (200 μg/ml) for another 24 h. Lactate production was measured by the lactate assay kit. *P < 0.05 vs. the indicated treatment.

Since HIF-1α has been reported as a glycolysis inducer^29^ and PDKs are regulators of the cellular energy metabolism shift, we attempted to investigate whether AGEs could enhance glycolysis during VSMC calcification through HIF-1α and PDK4. We first cultured VSMCs with AGE-BSA in calcium medium to detect lactate production and glucose consumption. Unexpectedly, the level of lactate, an important product of glycolysis, was decreased in a dose-dependent manner at different time points, and glucose consumption was also decreased in a dose-dependent manner at different time points (Fig. 6A). We next analyzed key enzymes of glucose metabolism; VSMCs were treated with AGE-BSA in the presence of calcium medium for 24 h, and qRT-PCR showed that hexokinase (HK), lactate dehydrogenase (LDH), isocitrate dehydrogenase (IDH), and glucose-6-phosphate dehydrogenase (G6PD) expression levels decreased as the concentration of AGE-BSA increased, but glucose 6-phosphatase (G6pase) levels did not change (Fig. 6B). To determine whether AGEs could influence the overall metabolism, the oxygen consumption rate (OCR) was also measured, and AGEs significantly inhibited OCR (Supplementary Figure 5). These results indicate that AGEs suppress glycolysis, aerobic oxidation, the pentose phosphate pathway, and mitochondrial respiratory capacity in VSMCs, leading to decreased glucose consumption and increased glycogen synthesis.

Despite the suppressive effect of AGEs on glucose metabolism, we still speculated that PDK4 knockdown could induce glycolysis activation; VSMCs transfected with PDK4 siRNA were used to detect lactate production. As shown in Fig. 6C, PDK4 knockdown accelerated AGE-BSA-down-regulated lactate production, which shows that AGEs and PDK4 have contradictory roles in the regulation of glucose metabolism.

## Discussion

This study demonstrated that AGEs increase the transcriptional and translational expression of HIF-1α and PDK4. Moreover, HIF-1α translocation and target gene expression were promoted by AGEs, and HIF-1α stabilization and nuclear translocation could regulate PDK4 expression. PDK4 knockdown by siRNA suppressed AGEs-induced VSMC calcification, as shown by RUNX2 protein levels, ALP activity, calcium deposition, and calcium nodule staining. In addition, we observed that AGEs suppressed glucose metabolism and that PDK4 siRNA silencing enhanced AGEs-down-regulated glycolysis. Taken together, we found that AGEs accelerate VSMC calcification through the HIF-1α/PDK4 signaling pathway and hinder glucose metabolism.

Vascular calcification and vascular events are strongly correlated^30^. Coronary artery calcification has been reported to be a predictor of adverse cardiac events in asymptomatic patients^31^. Oxidative stress, inflammation, apoptosis, and metabolic shifts contribute to vascular calcification through several downstream signaling cascades^12,32,33^. Recent clinical trials demonstrate that high fibroblast growth factor-23 (FGF-23) levels and high dose plus long-term statin therapy are also related to vascular calcification^34,35^. Based on previous reports, AGEs may be associated with the abovementioned vascular risks^12,32,33^; however, the role of AGEs in the pathogenesis of diabetic vascular calcification remains unclear.

Since HIF-1α has been demonstrated to be a glycolysis promoter^26,29^ and an important factor in phosphate-induced VSMC calcification^18^, we explored whether HIF-1α is involved in AGE-induced vascular calcification. We were the first to report that AGEs enhance HIF-1α transcriptional and translational expression in calcified VSMCs. In addition, AGEs induced HIF-1α translocation, further indicating the role of AGEs in transcriptional regulation. Several papers have shown that two important HIF-1α target genes, GLUT-1, a specific glucose transporter that hydrogen bonds with glucose as it moves through the membrane channel^36^, and VEGFA, which increases vascular permeability and angiogenesis^37^, induce osteoblastic differentiation and vascular calcification in different types of cells^38–40^. Our results demonstrated that GLUT-1 and VEGFA expression levels in VSMCs treated with AGEs were markedly increased compared with those in control VSMCs, further suggesting that HIF-1α and downstream genes participate in AGEs-induced VSMC calcification.

PDK4, an important mitochondrial matrix enzyme that controls metabolism shift, has been shown to be sensitive to intracellular ROS levels in our laboratory^12^. HIF-1α stabilization is also sensitive to cellular oxygen and mtROS^25^. Marycz K *et al*. reported that the oxidative stress/HIF-1α/PDK4 axis plays a key role in adipose stem cell (ASC) osteogenic differentiation^41^. AGEs were also shown to increase oxidative stress in VSMC calcification, as mentioned previously^11^. Therefore, whether AGEs-induced HIF-1α activation increases PDK4 expression levels in VSMC calcification is of interest. We found that HIF-1α stabilization or translocation up-regulated PDK4 expression, indicating that the AGEs/HIF-1α/PDK4 axis exists in VSMC calcification. In addition, PDK4 stimulation leads to mitochondrial dysfunction and excessive mtROS^14,42^; thus, a reciprocal loop among mtROS, HIF-1α, and PDK4 may be involved in AGEs-induced calcified VSMCs.

The relationship between PDK4 and vascular calcification has been previously elucidated^12,14^. This study further confirmed the role of PDK4 in AGEs-induced VSMC calcification. We discovered that PDK4 knockdown by siRNA reduced RUNX2 expression, which is a key factor for osteogenic gene expression^43^. ALP activity, calcium deposition, and calcified nodule formation were also attenuated after PDK4 inhibition. Our study shows for the first time that AGEs accelerate VSMC calcification through a PDK4-dependent pathway. However, PDK4 is associated with metabolic dysfunction, which leads to excessive ROS formation^13^. PDK4 inhibition may also down-regulate upstream signaling through a reciprocal loop, since HIF-1α and target genes are critical for vascular calcification^18,38–40^ as well as oxidative stress^11^. PDK4 interference may only partially alleviate VSMC calcification. Thus, it may be more accurate to conclude that AGEs accelerate VSMC calcification partly through a PDK4-dependent pathway.

Glucose metabolism plays an important role in vascular reactivity^44^, especially in VSMCs, which exhibit high glucose consumption and lactate production levels even under normal and well-oxygenated conditions^45^. Approximately 30% of the adenosine triphosphate (ATP) supply in VSMCs is derived from aerobic glycolysis, and 90% of the glycolysis flux contributes to lactate production^46^. During injury and atherogenesis, VSMCs will transdifferentiate from a contractile to a synthetic phenotype^47^. Lactate, the end product of glycolysis, has been reported to have an important role in promoting the synthetic phenotype in VSMCs^48^. Lactate can also induce osteoblast differentiation via HIF-1α^49^, and these findings demonstrate that enhanced glycolysis is important for VSMC phenotype differentiation and vascular function changes.

In VSMCs, PDK4 and HIF-1α are both promoters of glycolysis^12,26^; therefore, we speculated that AGEs may stimulate glycolysis. Unexpectedly, we found that AGEs significantly suppress lactate production and glucose utilization during VSMC calcification. In addition, expression levels of HK, LDH, IDH, and G6PD, which are related to glucose metabolism but not G6pase, and mitochondrial respiratory capacity as measured by OCR was decreased after treatment with AGEs. Although reduced G6PD expression may be due to the inhibition of glucose-6-phosphate, and the down-regulation of IDH expression may be attributed to mitochondrial dysfunction caused by PDK4 activation^13^, all these findings suggest that AGEs inhibit glycolysis and impair normal mitochondrial function during VSMC calcification. A recent report has suggested that during atherogenesis, VSMCs have increased mitochondrial dysfunction and use of glycolysis; enhanced glycolysis could be a compensatory response to energetic failure^50^. In our study, AGEs induced mitochondrial dysfunction during VSMC calcification as measured by the expression of key enzymes of Kreb’s cycle and by OCR. Mitochondrial dysfunction may be associated with AGEs-induced oxidative stress, leading to mitochondrial membrane potential decline and an impaired mitochondrial respiratory chain^51,52^. However, why AGEs inhibit the glycolysis response are still unclear, although in human umbilical vein endothelial cells (HUVECs), glycolysis also declined after AGEs exposure^51^. PDK4 activation elevated the lactate concentration in VSMC supernatant in our experiments, a possible explanation might be that AGEs suppress glycolysis as a whole, counteracting the effects from HIF-1α and PDK4. Further investigation of the basic mechanisms of the AGEs-induced glucose metabolism shift will be critical for treatment of diabetic complications.

Our study also has some limitations. A calcification medium composed of 0.25 mmol/L L-ascorbic acid and 10^−8^ M dexamethasone in addition to β-GP is a conventional calcification medium^53^, and our results suggested that VSMCs treated with conventional calcification medium calcify more easily than those treated with β-GP alone (Supplementary Figure 6). Therefore, the conventional calcification medium is a better choice for further research. In addition, although HIF-1α has been reported to bind the PDK4 promoter in results of a luciferase reporter assay^22^, chromatin immunoprecipitation could better prove the direct interaction.

In summary, this study demonstrates that AGEs enhance vascular calcification through the HIF-1α/PDK4 pathway. In addition, glucose metabolism is suppressed during AGE-induced VSMC calcification. Therefore, inhibition of the AGEs/HIF-1α/PDK4 pathway might be an effective approach for the prevention of diabetic vascular calcification. However, the basic mechanisms of the AGEs-mediated glucose metabolism shift remain to be investigated in depth.

## Materials and Methods

### Ethics statement

All animal studies were approved by the Ethics Committee of Southeast University and were performed in accordance with the guidelines for the care and use of laboratory animals published by the China National Institutes of Health.

### Materials

Deferoxamine mesylate salt (DFOM) (D9533), dichloroacetic acid (DCA) (D54702), β-glycerophosphate disodium salt hydrate (β-GP) (G5422), bovine serum albumin (BSA) (A1933), and D-glucose (G7528) were provided by Sigma-Aldrich (Saint Louis, USA). 2-Methoxyestradiol (2-MeOE2) (S1233) was obtained from Selleck (Texas, USA). The calcium assay kit (C004-2), alkaline phosphatase (ALP) activity kit (A059-2), glucose assay kit (F006), and lactate assay kit (A019-2) were obtained from the Nanjing Jiancheng Bioengineering Institute (Nanjing, China). The Alizarin red S staining kit (0223) was purchased from Shunbai Biologicals Inc. (Shanghai, China). The bicinchoninic acid (BCA) protein assay kit (P0009) and CCK-8 assay kit (C0037) were provided by Beyotime Biotechnology (Jiangsu, China). The antibody against HIF-1α (CST36169) was obtained from Cell Signaling Technology (Danvers, MA, USA). Anti-PDK4 (ab89295) and anti-RUNX2 (ab76956) antibodies were purchased from Abcam (Cambridge, MA, USA). Anti-β-actin (BL005B) and all secondary antibodies were provided by Biosharp (Anhui, China).

### Cell culture

Primary VSMCs were isolated from six-week-old Sprague Dawley rat thoracic aortas (Experimental Animal Centre, Southeast University, Nanjing, China) according to previous protocols^54^. VSMCs between passages 4 and 8 were cultured in a 1:1 mixture of Dulbecco’s Modified Eagle’s Medium (DMEM) and Ham’s F12 medium with 10% fetal bovine serum and antibiotics at 37 °C with 5% CO_2_. VSMC calcification was induced in DMEM containing 1% FBS in the presence of 10 mM β-GP, and the culture media was replaced twice per week.

### Preparation of AGE-bovine serum albumin (BSA)

One gram of BSA and 3 g of D-glucose were dissolved in 10 mL of sodium phosphate buffer (PBS). The solution was incubated at 37 °C for 90 days in the dark and then dialyzed against PBS. As a control, BSA was incubated without D-glucose. The AGE-BSA concentration was estimated by a BCA protein assay kit.

### Cell viability analysis

VSMCs were seeded onto 96-well plates at a density of 5,000 cells/well for 24 h. Then, VSMCs were incubated with 0, 50, 100, 200 and 400 μg/ml AGE-BSA in the presence of 10 mM β-GP for 12, 24, 48, and 72 h. After treatment, 10 μL of CCK-8 was added to each well and incubated for 2 h at 37 °C. Absorbance was measured at a wavelength of 450 nm.

### Alizarin red S staining

VSMCs were fixed in 4% paraformaldehyde for 30 min at room temperature, washed twice with PBS, and then stained with 1% Alizarin red S (pH 8.4) for 30 min at 37 °C. Then, excess Alizarin red S reagent was removed by washing twice with PBS. The calcium nodules were observed under a microscope.

### Measurement of calcium content

VSMCs were decalcified with 0.6 M HCl for 24 h at 37 °C, and then, cells were washed three times with PBS and solubilized with 0.1 M NaOH containing 0.1% SDS. The calcium content in VSMCs was measured using the calcium assay kit and normalized to the total protein content with the BCA protein assay kit.

### ALP activity assay

VSMCs were solubilized with RIPA lysis buffer. After centrifugation, the supernatants were examined by the ALP activity kit and normalized to total protein content with the BCA protein assay kit.

### Measurement of lactate production and glucose consumption

The supernatants collected from cultured VSMCs were examined by the lactate assay kit and glucose assay kit according to the manufacturer’s instructions.

### Small interfering RNA transfection

Small interfering RNA (siRNA) was designed by Obio Technology (Shanghai, China). The sequences of the PDK4 siRNA were as follows: sense, 5′-GGATTACTGACCGCCTCTT-3′; and antisense, 5′-AAGAGGCGGTCAGTAATCC-3′. The sequences of the negative control siRNA were as follows: sense, 5′-TTCTCCGAACGTGTCACGT-3′; and antisense, 5′-ACGTGACACGTTCGGAGAA-3′. The siRNAs were transfected into cells with Lipofectamine 2000 (Invitrogen Life Science, Grand Island, NY) according to the manufacturer’s protocol. The transfection efficiency was examined by western blotting.

### Immunofluorescence staining

VSMCs were fixed with 4% paraformaldehyde, permeabilized with 0.1% Triton X-100 for 20 min, and then blocked with 5% BSA for 0.5 h at room temperature. Primary antibody (anti-HIF-1α, 1:200) was incubated with cells overnight at 4 °C, and then, the cells were incubated with appropriate second antibodies for 0.5 h in the dark. Nuclei were stained with DAPI for 15 min. The images were visualized using a confocal microscope (FV10i, Olympus, Japan).

### Real-time qRT-PCR

Total RNA was isolated using TRIzol according to the manufacturer’s instructions. RNA purity was evaluated based on the A260/A280 ratio using a Merinton SMA4000. Reverse transcription (RT) was performed with Prime Script TM Master Mix (Takara). Quantitative reverse transcriptase–polymerase chain reaction (qRT-PCR) was performed on a StepOne Plus system (ABI) using SYBR Green Mix. PCR primers are shown in Table 1. Results were normalized to the expression level of β-actin.

**Table 1:** Primer sequences for the qRT-PCR analysis.

Genes	Primer sequences
PDK4	Forward, 5′-AGGGAGGTCGAGCTGTTCTC-3′
	Reverse, 5′-GGAGTGTTCACTAAGCGGTCA-3′
HIF-1α	Forward, 5′-ACCTTCATCGGAAACTCCAAAG-3′
	Reverse, 5′-ACTGTTAGGCTCAGGTGAACT-3′
GLUT-1	Forward, 5′-TCTCGGCTTAGGGCATGGAT-3′
	Reverse, 5′-TCTATGACGCCGTGATAGCAG-3′
VEGFA	Forward, 5′-TGGATGTCTACCAGCGAAGC-3′
	Reverse, 5′-ACGCACTCCAGGGCTTCA-3′
HK	Forward, 5′-GGAGGCGAGAACATCAAGCC-3′
	Reverse, 5′-CGGCCTTCCCTCGTAGTGA-3′
LDH	Forward, 5′-CGGTCAAGGAGAGGAGCTTAC-3′
	Reverse, 5′-GGACTAGCCCTCGCTTATCTTT-3′
IDH	Forward, 5′-GGAGAAGCCGGTAGTGGAGAT-3′
	Reverse, 5′-GGTCTGGTCACGGTTTGGAA-3′
G6 Pase	Forward, 5′-CGACTCGCTATCTCCAAGTGA-3′
	Reverse, 5′-GTTGAACCAGTCTCCGACCA-3′
G6PD	Forward, 5′-CACAGTGGACGACATCCGAAA-3′
	Reverse, 5′-AGCTACATAGGAATTACGGGCAA-3′
β-actin	Forward, 5′-GGCTGTATTCCCCTCCATCG-3′
	Reverse, 5′-CCAGTTGGTAACAATGCCATGT-3′

### Western blot analysis

VSMCs were lysed with RIPA lysis buffer containing protease inhibitors for 30 min at 4 °C. After centrifugation, the supernatants were harvested, and the protein concentration was measured using the BCA protein assay kit. Subsequently, 60 μg of total protein was loaded onto an SDS-PAGE gel and then transferred onto nitrocellulose membranes. The membranes were blocked with 5% non-fat milk for one hour. Subsequently, the membranes were incubated with different primary antibodies (HIF-1α: 1:1000, PDK4: 1:2000, RUNX2: 1:1000, β-actin: 1:2000) overnight at 4 °C and then visualized using anti-rabbit IgG (1:5000) conjugated with horseradish peroxidase for 1 h at room temperature. The blots were detected using ECL, and the results were quantified by Image-Pro Plus 6.0 software and then normalized to β-actin.

### Statistical analysis

All experiments were independently repeated at least three times. All data are presented as the mean ± standard deviation (SD). Statistical analyses were performed using Statistical Package for Social Science (SPSS) 22.0 software (SPSS, Chicago, IL, USA). Data were plotted using GraphPad Prism software (GraphPad Prism 7.0; GraphPad Software Inc., La Jolla, CA). Student’s t-test was used to compare two variables, and one-way analysis of variance (ANOVA) was used to compare more than two groups. All statistical tests were two-tailed, and all data followed a normal distribution. Values of *P* < 0.05 were considered to be statistically significant.

## Electronic supplementary material

Supplementary information
