# Supplementary material for: Advanced glycation end products accelerate calcification in VSMCs through HIF-1α/PDK4 activation and suppress glucose metabolism
Source: Sci Rep. 2018 Sep 13;8:13730. doi: 10.1038/s41598-018-31877-6 (PMC6137084; doi:10.1038/s41598-018-31877-6)
Supplement: Supplementary file 1 — Supplementary information [file 41598_2018_31877_MOESM1_ESM.docx]

**Advanced glycation end products accelerate calcification in VSMCs through** **HIF-1α/PDK4 activation and suppress glucose metabolism**

Yi Zhu^1^, Wen-Qi Ma^1^, Xi-Qiong Han^1^, Ying Wang^1^, Xin Wang^1^, and Nai-Feng Liu^1,^**^*^**

^1^Department of Cardiology, Zhongda Hospital, School of Medicine, Southeast University, Nanjing 210009, P.R. China.

**Correspondence to:** Nai-Feng Liu, **email:** [liunf@seu.edu.cn](mailto:liunf@seu.edu.cn)

**Oxygen consumption rate (OCR) assay**

VSMCs were seeded into Seahorse 24-well plates at 8000 cells/well and were then treated as previously reported ^[1](#_ENREF_1)^. OCR was measured by a Seahorse XFe24 analyzer (Seahorse Bioscience, Boston, MA, United States). A Mitostress kit (Seahorse Bioscience), oligomycin, carbonylcyanide 4-(trifluoromethoxy) phenylhydrazone (FCCP) and rotenone were used according to the manufacturer’s instructions. All measurements were normalized to the cell number. Basal respiration = (last rate measurement before first injection)−(minimum rate measurement after antimycin injection). Maximal respiration = (maximum rate measurement after FCCP injection)−(minimum rate measurement after antimycin injection).

**Cytoplasmic and nuclear protein extraction**

VSMCs were lysed using a nuclear and cytoplasmic protein extraction kit (P0027) purchased from Beyotime Biotechnology (Jiangsu, China) according to the manufacturer’s instructions.


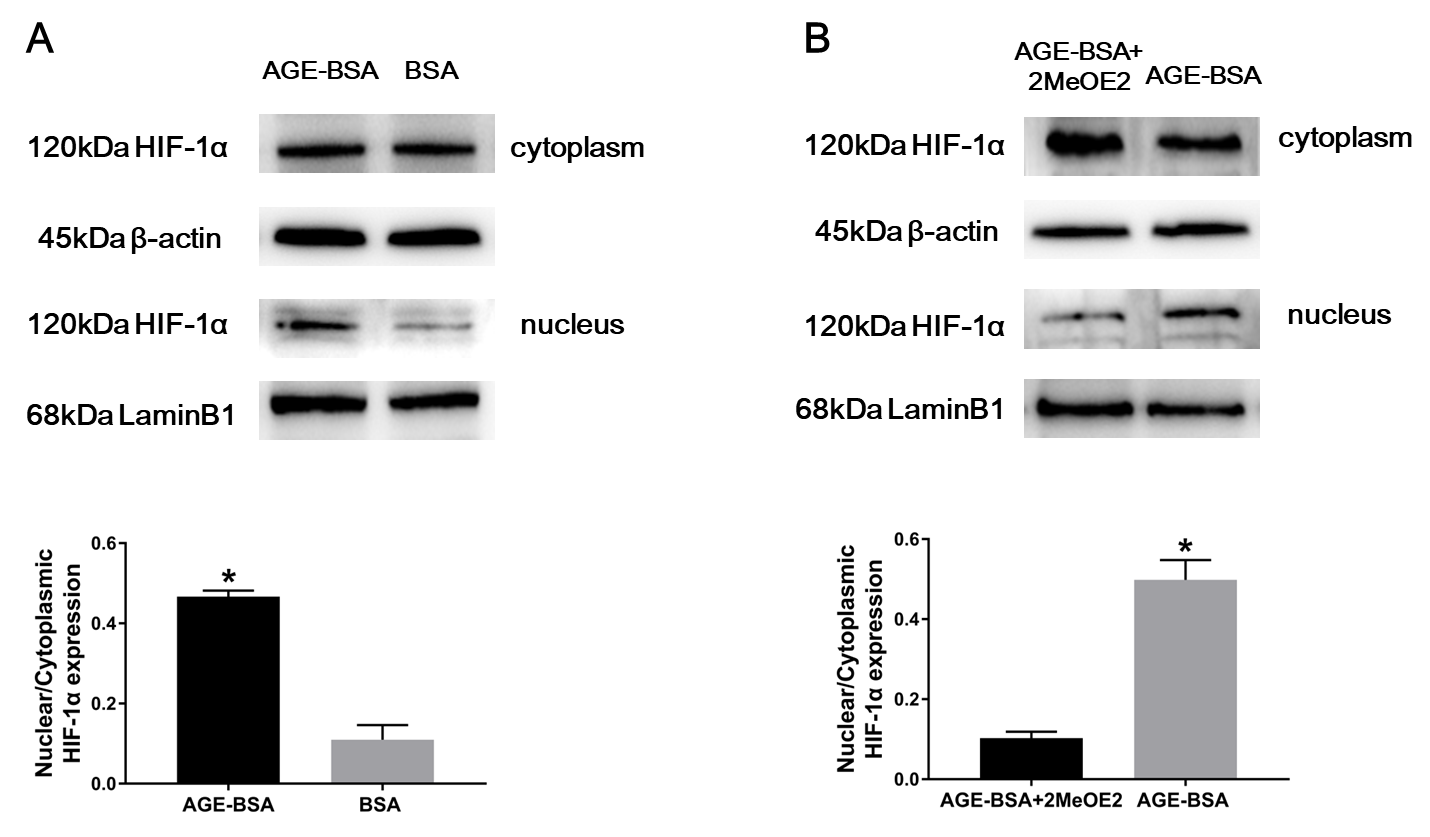


**Supplementary Figure 1. AGEs induced HIF-1α nuclear translocation. (A)** Nuclear vs cytoplasmic HIF-1α expression in calcified VSMCs after AGE-BSA (200 μg/ml) treatment was detected by western blotting. * *P* < 0.05 compared with the BSA group. **(B)** Calcified VSMCs were pretreated with 2-MeOE2 (10 μM) for 2 h and then incubated with AGE-BSA (200 μg/ml) for 24 h. HIF-1α expression was measured by western blotting. * *P* < 0.05 compared with the AGE-BSA+2MeOE2 group.


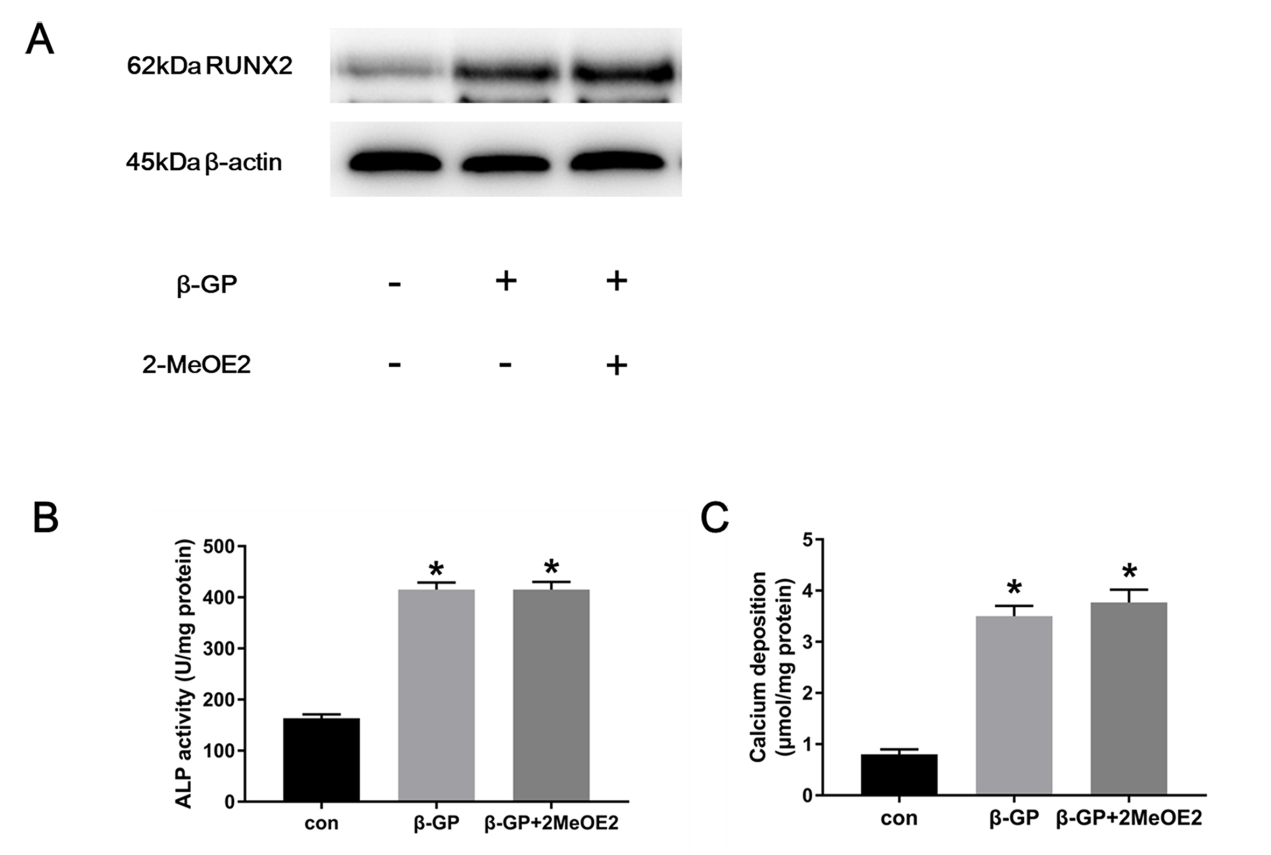


**Supplementary Figure 2. 2-MeOE2 had no influence on VSMC calcification. (A)** After β-GP (10 mM) and 2-MeOE2 (10 μM) exposure, RUNX2 expression in VSMCs was detected by western blotting. **(B, C)** VSMCs were cultured in the presence of β-GP (10 mM) with or without 2-MeOE2 (10 μM) for 7 days, and ALP activity and calcium deposition were detected. * *P* < 0.05 compared with the normal control group.


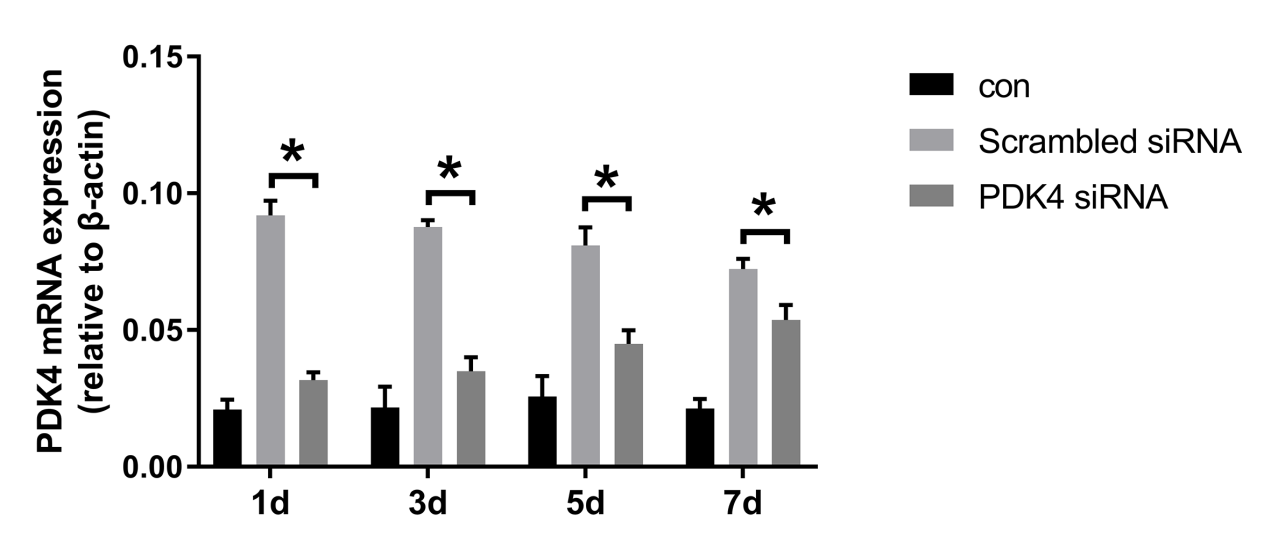


**Supplementary Figure 3. Analysis of PDK4 siRNA transfection efficiency within 7 days.** PDK4 siRNA transfection efficiency was measured by qRT-PCR. * *P* < 0.05 vs. the indicated treatment.


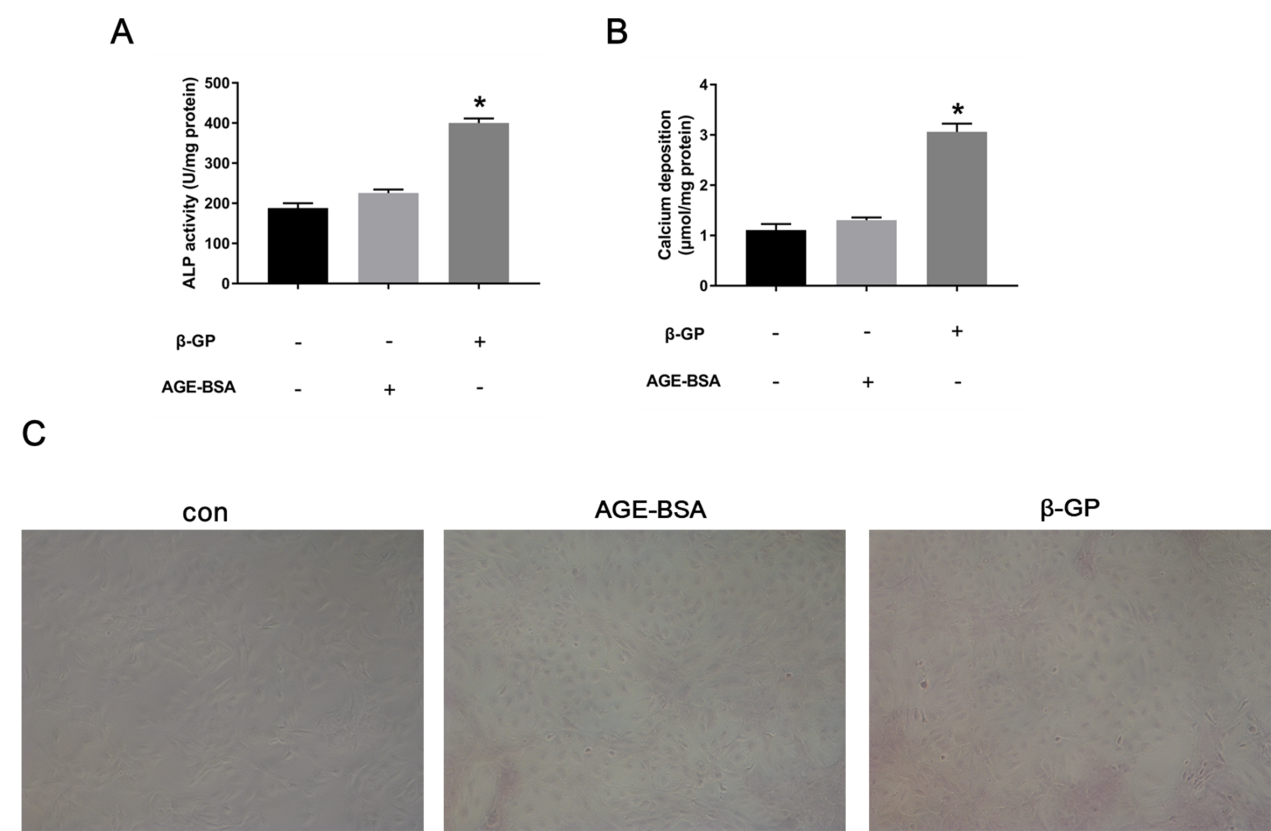


**Supplementary Figure 4. AGEs treatment alone was not an obvious cause of VSMC calcification. (A, B)** VSMCs were cultured with β-GP (10 mM) or AGE-BSA (200 μg/ml) for 7 days, and ALP activity and calcium deposition were detected. * *P* < 0.05 compared with the normal control group. **(C)** After 21 days AGE-BSA (200 μg/ml) or β-GP (10 mM) treatment, calcium nodule formation was visualized by Alizarin red S staining.


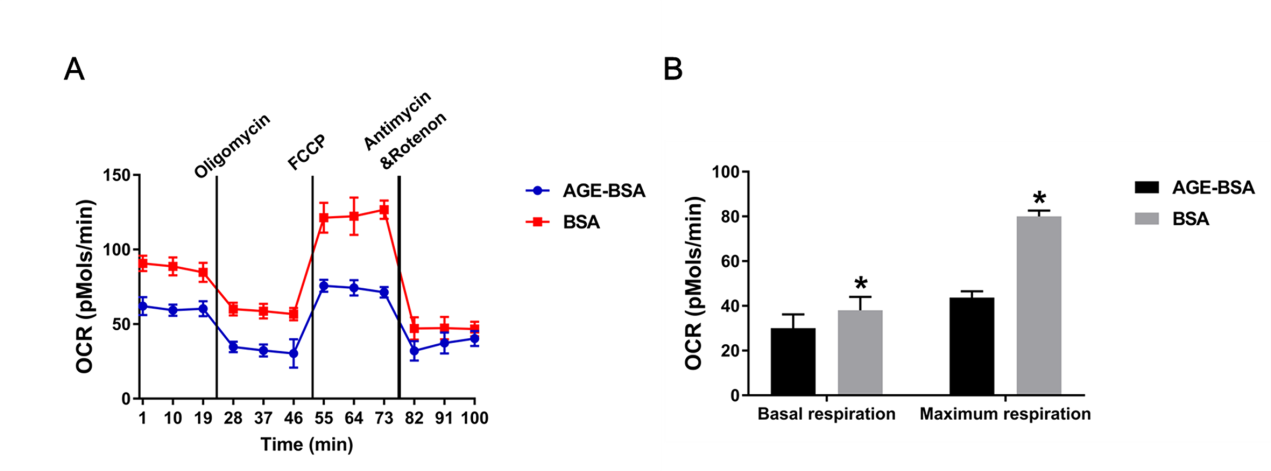


**Supplementary Figure 5. AGEs suppressed** **mitochondrial respiratory capacity. (A)** OCR in VSMCs after AGE-BSA (200 μg/ml) or BSA (200 μg/ml) treatment for 24 h was detected by a Seahorse XFe24 analyzer. **(B)** We also measured the basal respiratory and maximum respiratory capacities. * *P* < 0.05 compared with the AGE-BSA group.


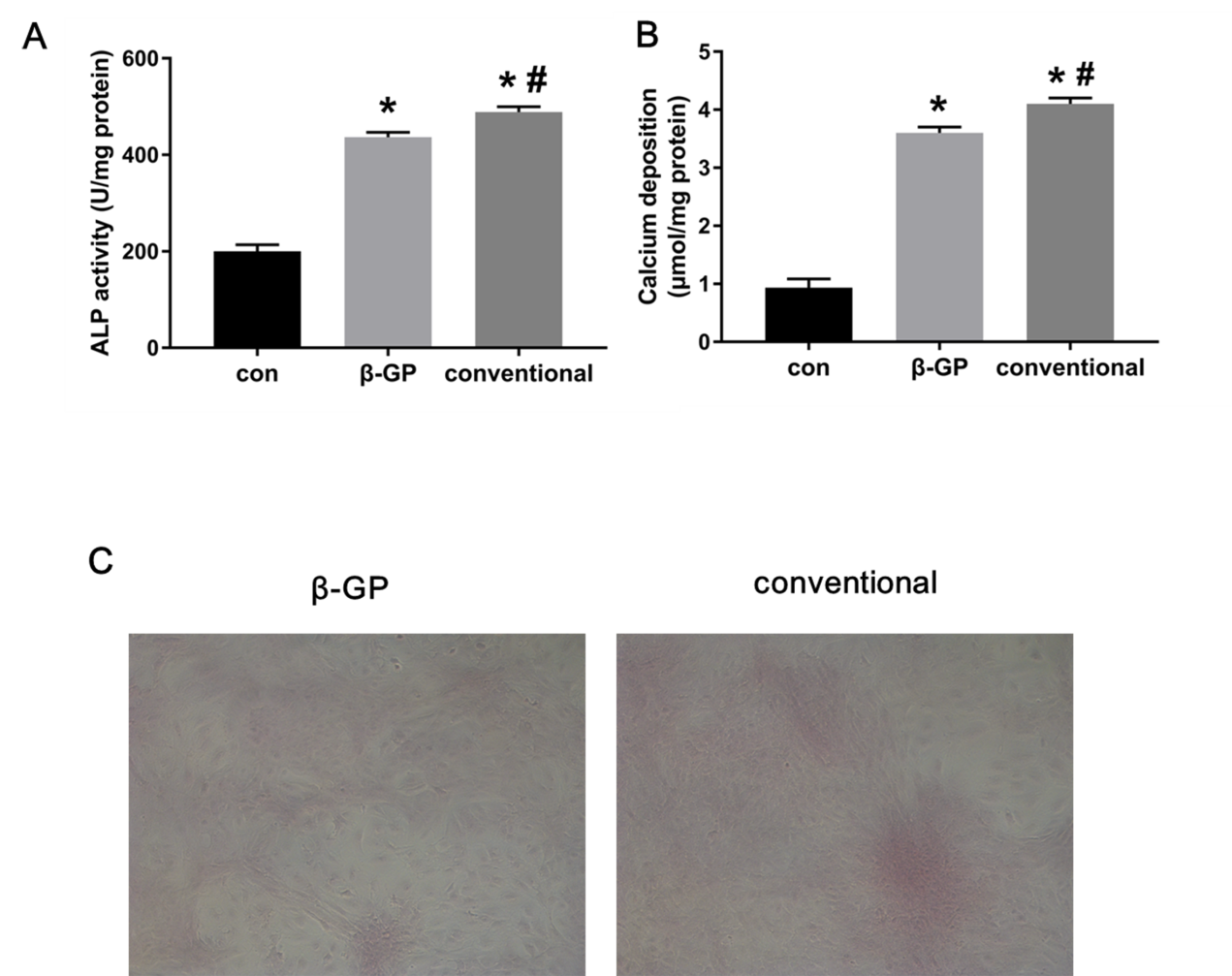


**Supplementary Figure 6. Conventional calcium medium contributed to VSMC calcification more easily. (A, B)** VSMCs were cultured with β-GP (10 mM) or conventional calcium medium for 7 days, and ALP activity and calcium deposition were detected. * *P* < 0.05 compared with the normal control group. # *P* < 0.05 compared with the β-GP group. **(C)** After 21 days of β-GP (10 mM) or conventional calcium medium treatment, calcium nodule formation was visualized by Alizarin red S staining.

**References**

1 Makela, J. *et al.* Peroxisome proliferator-activated receptor-gamma (PPARgamma) agonist is neuroprotective and stimulates PGC-1alpha expression and CREB phosphorylation in human dopaminergic neurons. *Neuropharmacology* **102**, 266-275, doi:10.1016/j.neuropharm.2015.11.020 (2016).

**Supplementary Figure 1A**


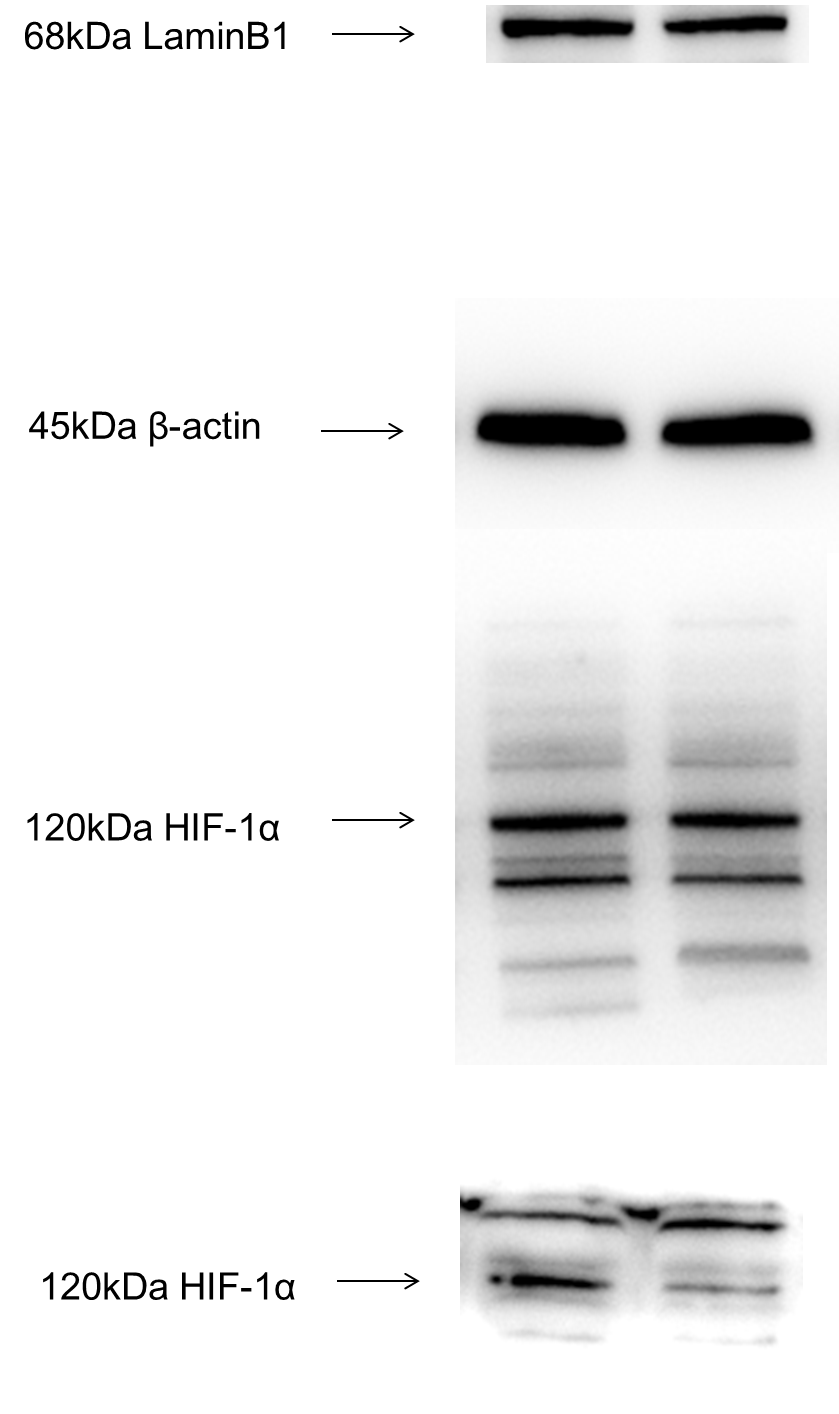


**Supplementary Figure 1B**

**
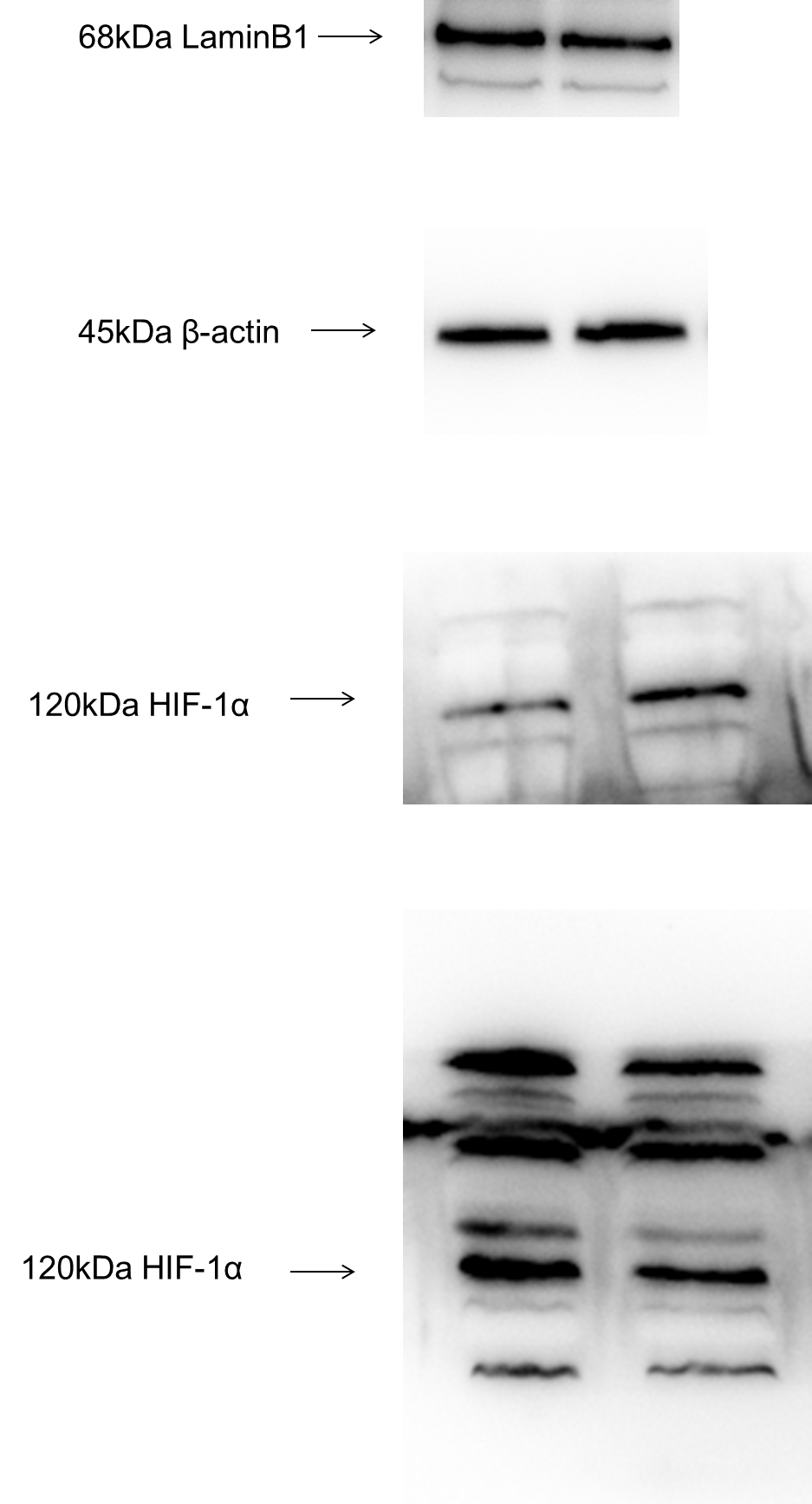
**

**Supplementary Figure 2A**

**
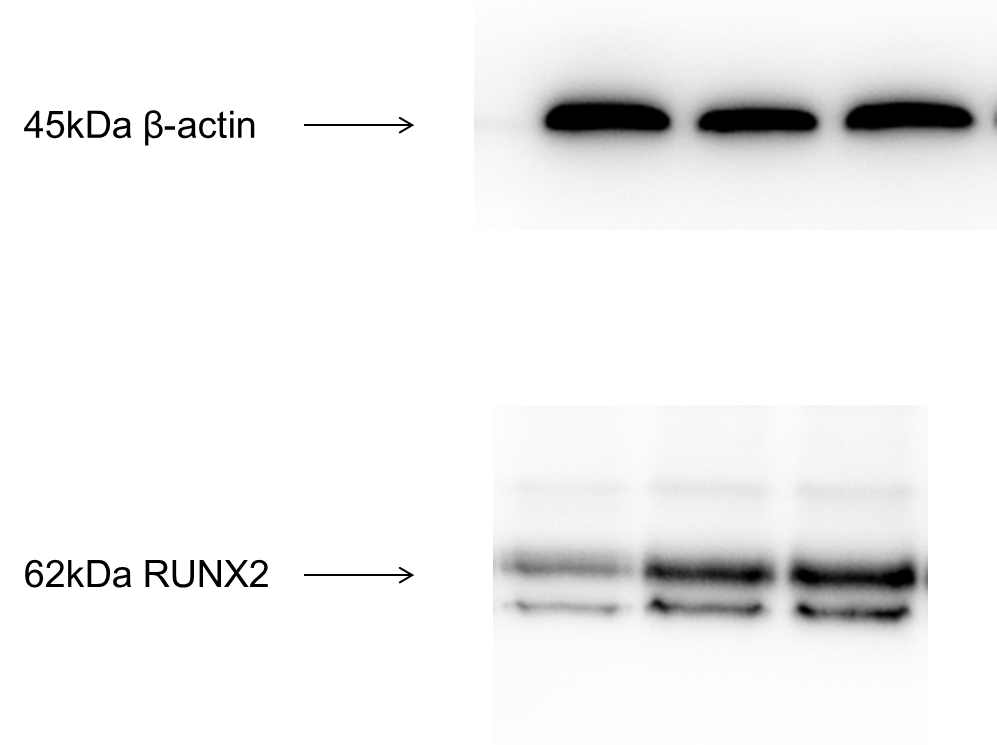
**
